# Case Report: *Talaromyces marneffei* Infection in a Chinese Child With a Complex Heterozygous *CARD9* Mutation

**DOI:** 10.3389/fimmu.2021.685546

**Published:** 2021-06-21

**Authors:** Hongjun Ba, Huimin Peng, Liangping Cheng, Yuese Lin, Xuandi Li, Xiufang He, Shujuan Li, Huishen Wang, Youzhen Qin

**Affiliations:** ^1^ Department of Paediatric Cardiology, Heart Centre, The First Affiliated Hospital, Sun Yat-sen University, Guangzhou, China; ^2^ Key Laboratory on Assisted Circulation, Ministry of Health, Guangzhou, China

**Keywords:** *Talaromyces marneffei* infection, *CARD9* mutation, fungal immunodeficiency, CARD9 deficiency, immunodeficiency disease

## Abstract

*Talaromyces marneffei* (TM) infection is rarely seen in clinical practice, and its pathogenesis may be related to deficiency in antifungal immune function. Human caspase recruitment domain-containing protein 9 (CARD9) is a key molecule in fungal immune surveillance. There have been no previous case reports of TM infection in individuals with *CARD9* gene mutations. Herein, we report the case of a 7-month-old Chinese boy who was admitted to our hospital with recurring cough and fever with a papular rash. A blood culture produced TM growth, which was confirmed by metagenomic next-generation sequencing. One of the patient’s sisters had died of TM septicaemia at 9 months of age. Whole exome sequencing revealed that the patient had a complex heterozygous *CARD9* gene mutation with a c.1118G>C p.R373P variation in exon 8 and a c.610C>T p.R204C variation in exon 4. Based on the culture results, voriconazole antifungal therapy was administered. On the third day of antifungal administration, his temperature dropped to within normal range, the rash gradually subsided, and the enlargement of his lymph nodes, liver, and spleen improved. Two months after discharge, he returned to the hospital for a follow-up examination. His general condition was good, and no specific abnormalities were detected. Oral voriconazole treatment was continued. Unexplained TM infection in HIV-negative individuals warrants investigation for immune deficiencies.

## Introduction

*Talaromyces marneffei* (TM) is an opportunistic pathogen. TM is a member of the family Trichocomaceae, order Eurotiales, class Eurotiomycetes, division Ascomycota. It is the only member of the genus Talaromyces and is considered an important human pathogen. TM infection is clinically rare and occurs mainly in individuals with HIV infection. In HIV-negative individuals, TM infection occurs mainly in patients with congenital immune deficiencies (1). Many genetic conditions are associated with congenital immune deficiencies, including *CARD9* (caspase recruitment domain-containing protein 9) gene mutations. Human *CARD9* is a key molecule in fungal immune surveillance (2). In the year 2013, the primary genetic aetiology of deep dermatomycosis was identified as homozygous loss of function mutations in *CARD9* in 17 patients from eight unrelated North African families (3). One homozygous premature stop codon mutation (Q289*) was found in 15 patients from seven unrelated families in Algeria and Tunisia, and one homozygous missense mutation (R101C) was found in two Moroccan siblings (4). The CARD9Q289* mutation was also recently identified in an Egyptian patient with extensive skin and nail mycosis (5). Experiments in mice have shown that CARD9 is an adaptor molecule, expressed primarily in macrophages and myeloid dendritic cells, and plays a central role in antifungal defence by receiving signals from several C-type lectin-like receptors and stimulating proinflammatory responses (6). In addition, CARD9-deficient cells have been shown to exhibit selective impairment of tumour necrosis factor-α and interleukin-6 production when stimulated by fungal antigens (6–8). Several cases of *CARD9* mutations combined with fungal infections such as candidiasis have been reported, but there are no previous reports of *CARD9* gene mutation combined with TM infection. In this report, we describe an infant with *CARD9* mutation combined with TM infection in order to raise clinicians’ awareness of *CARD9* mutations as a possible cause of vulnerability to fungal infections.

## Case Description

A 7-month-old Chinese boy was admitted to our hospital with a 2-month history of a recurring cough, fever for nine days, and a worsening rash for two days. The local hospital had administered a variety of antimicrobials prior to referral, but the patient’s condition had not improved. Physical examination on admission revealed facial and bodily maculopapules, reddish-brown papules covering the limbs, swelling of the lower limbs, cervical lymphadenopathy, and faint wet rales on auscultation of both lungs. His heart rate was 160–170 bpm with normal heart sounds. Hepatosplenomegaly was present, and neurological examination was normal. He had previously been observed to have left axillary lymph node enlargement after BCG inoculation at birth. The patient was the third child in the family and had two older sisters. The second sister had died of recurrent fever with severe sepsis at 9 months of age, with TM produced in bone marrow and blood cultures.

Chest computed tomography (CT) examination revealed bilateral pulmonary infiltrates. Abdominal CT revealed significant hepatosplenomegaly and multiple enlarged abdominal lymph nodes. Colour Doppler ultrasound of the heart showed enlargement of the left ventricle and a small pericardial effusion, but no significant dilation of the left and right coronary arteries.

Laboratory tests revealed a serum C-reactive protein (CRP) level of 272 mg/L (reference range, <10 mg/L) and serum procalcitonin (PCT) level of 2.73 ng/mL (reference range, <0.05 ng/mL). Routine haematologic testing revealed the following: white blood cell count (WBC), 33×10^9^ cells/L (reference range, 4–10×10^9^ cells/L); neutrophil count, 16.13×10^9^ cells/L; lymphocyte count, 12.85×10^9^ cells/L; and monocyte count, 1.24×10^9^ cells/L. The serum N-terminal pro b-type natriuretic peptide level was 979.2 pg/mL (reference range, 0–84 pg/mL). Serological tests for HIV were negative. Cellular immune function tests revealed CD3+, 82.9%; CD3+CD4+, 50.8%; CD3+CD8+, 29.8%; CD19+, 12.8%; and natural killer cells, 4%. Results of humoral immune function testing were within normal limits. Th1/Th2/Th17 cytokine detection showed the following: tumour necrosis factor level, 1.48 pg/mL (reference range, 0–4.6 pg/mL); interleukin-6 level, 4.6 pg/mL (reference range, 0–5.3 pg/mL); interferon-gamma level, 2.66 pg/mL (reference range, 0–7.42 pg/mL); interleukin-17 level, 6.49 pg/mL (reference range, 0–20.6 pg/mL); interleukin-10 level, 8.59 pg/mL (reference range, 0–4.91 pg/mL); and interleukin-2 level, 1.11 pg/mL (reference range, 0–5.71 pg/mL). A blood culture produced growth of TM. No growth was found on bone marrow microbial culture.

**Figure 1:**
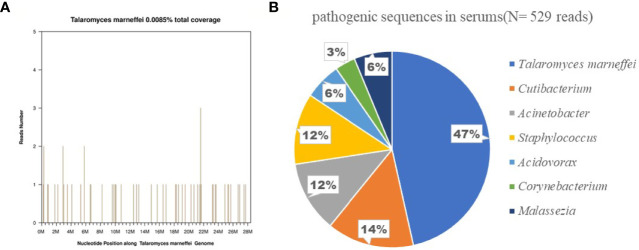
Confirmation of *talaromyces marneffei* specific amplification from plasma by next-generation sequencing. **(A)** shows the reads mapped to talaromyces marneffei derived from NGS data. A total of 248 reads mapped to *talaromyces marneffei* in the reference database which contains about 8000 pathogen genomes, and got a total coverage of 0.0085% respectively. **(B)** shows the distribution of bacterial sequences (N = 529 reads) identified in the patient’s plasma included *Talaromyces marneffei* (N = 248;47%), *Cutibacterium, Acinetobacter, Staphylococcus, Acidovorax, Corynebacterium, Malassezia*.

Metagenomic next-generation sequencing (mNGS) of pathogenic blood microorganisms confirmed TM infection (**Figure 1**). Bone marrow cytology and culture showed no obvious abnormalities.

**Figure 2:**
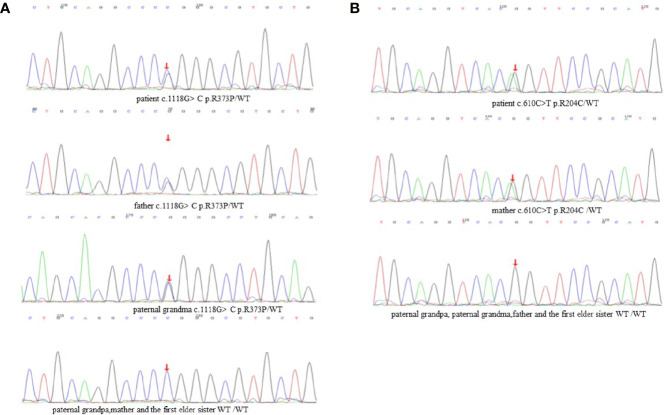
Family evaluation of *CARD9*. **(A)** c.1118G>C p.R373P variation in exon 8. **(B)** c.610C>T p.R204C variation in exon 4.

**Figure 3:**
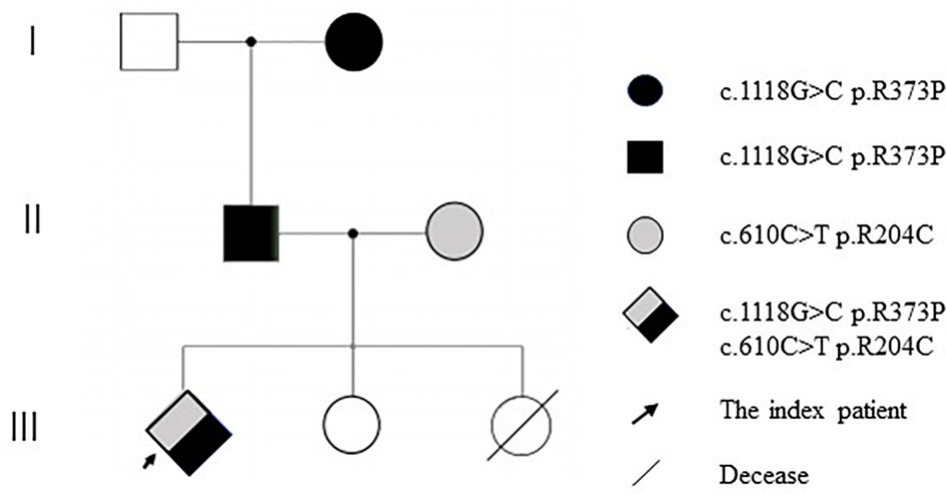
Pedigree of *CARD9* mutations (Due to death, no genetic analysis was performed for the second elder sister).

DNA samples from the patient’s family members were collected with their consent (**Figure 2**). *CARD9* was amplified using specific primers. The patient was found to have a complex heterozygous *CARD9* genotype with a c.1118G>C p.R373P variation in exon 8 and a c.610C>T p.R204C variation in exon 4 (**Figure 3**). The Sanger method was used to verify the results. The patient’s father and paternal grandmother both had the c.1118G>C p.R373P variation in exon 8, but neither fell ill. In addition, the patient’s mother had a c.610C>T p.R204C variation in exon 4 but had no medical history of note(**Figure 3**). Sorting Intolerant From Tolerant (SIFT) is a tool for predicting the harm of genetic variation. The SIFT score ranges from 0 to 1, and a score <0.05 is predicted to be deleterious. The SIFT scores of variations c.1118G>C p.R373P and c.610C>T p.R204C were 0.007 and 0, respectively. The SIFT prediction hints were both damaging. The *CARD9* gene mutation with a c.1118G >C p.R373P variation in exon 8 has been reported previously, but the c.610C>T p.R204C variation in exon 4 has not.

Other pathogens detected by mNGS were considered as background organisms due to low copy numbers. Because the local hospital had administered potent anti-bacterial treatment with no results, we considering the likelihood of bacterial infection to be low; therefore, at first, we administered only cefuroxime as an empirical treatment. We administered voriconazole antifungal therapy based on the TM culture susceptibility testing results. On the third day of administration, the patient’s body temperature returned to normal, rash gradually subsided, lymphadenopathy and hepatosplenomegaly improved, and heart rate returned to normal. After seven days of voriconazole treatment, his WBC and serum levels of CRP, PCT, and NT-proBNP were all within normal limits.

Two months after discharge, the patient returned to the hospital for a follow-up examination. His general condition was normal without any specific abnormalities. The treatment with oral voriconazole was continued.

## Discussion

TM infection is endemic in tropical regions, especially Thailand, Vietnam, northeastern India, Southern China, Hong Kong, Taiwan, Laos, Malaysia, Myanmar, Cambodia and Laos (9). TM is a deep pathogenic fungus first isolated by Capponi et al. in 1956 from the liver of the Vietnamese bamboo mouse (10). The bamboo mouse carries TM, and its excrement travels through water to contaminate soil. Humans can be infected through the skin, the respiratory tract, or the gastrointestinal tract. In the past, TM infection has been recognized as one of the three major opportunistic infections of AIDS, along with tuberculosis and cryptococcosis (11). In recent years, improved treatment of HIV infection through aggressive antiretroviral therapy and other measures to control the HIV/AIDS epidemic have led to changes in the epidemiology of TM infection and an increasing number of non-HIV infected patients with other immunocompromised conditions. CARD9 mutation is one of the important causes of primary immunodeficiency disease (8).There have been no reports of TM infection patients with CARD9 mutations in children.

TM can invade multiple organs of the body, proliferate in macrophages, and spread through the endothelial network, especially in the blood, bone marrow, skin, lungs, and reticuloendothelial tissue (12). Similar to other intracellular pathogens, T-lymphocyte-derived cytokines activate macrophages, especially those that respond to Th1, such as interleukin (IL)-12, IFN-γ, and tumor necrosis factor (TNF)-α, which are important cytokines for host defense against TM infection (13). This is supported by the observation that in mice with reduced T lymphocytes, TM infection is always fatal, while in healthy mice the fungus can be cleared within three weeks (14, 15). There was no significant increase in TNF and IL in this patient, so the clinical presentation was very severe. The patient presents with high fever, rash, hepatosplenomegaly, and cervical lymphadenopathy. These were consistent with the possible mechanisms of TM infection reported in the above literature.

With general bacterial infectious diseases, serum CRP and PCT levels will be significantly increased, and in fungal infectious diseases, they are rarely increased. However, when fungal sepsis occurs, serum CRP and PCT levels will significantly increase. This suggests that in clinical practice, in addition to bacterial infection, the possibility of fungal sepsis should be considered for patients with significantly abnormal serum CRP and PCT levels. Due to significantly elevated CRP and PCT in our patient, the local hospital provided empirical anti-bacterial treatment with vancomycin and meropenem, but the effect was not ideal, and recurrent high fever persisted. Therefore, fungal sepsis was suspected. Further mNGS identified the pathogen as TM, and the patient improved after antifungal treatment with voriconazole. Therefore, in children with fever of unknown origin and multi-system infection in regions with high incidence of TM infection, TM should be considered in the differential diagnosis when screening for fungal infection. TM is the only rare pathogen with temperature biphasic type in *Penicillium*. Routine cultures are prone to false negatives, and mNGS is a new technology for detection of pathogens using metagenomic next-generation sequencing. mNGS can be used for in-depth and rapid identification of pathogens without culture, which is more sensitive than traditional culture methods. Therefore, Pathogens should be identified as soon as possible using mNGS so that effective targeted treatment can be initiated.

TM infection is relatively rare clinically and mainly occurs in patients with acquired immunodeficiency. It has been reported that 30% of patients with AIDS are infected with TM (16). However, our patient repeatedly tested HIV-negative, and the history of an infant sister who had died due to TM infection suggested a possible genetic immunodeficiency in the patient’s family. Therefore, we further conducted family medical whole exon gene sequencing, and the test indicated that the child had a compound heterozygous *CARD9* gene mutation.

*CARD9*, a member of the *CARD* family, is an important linking protein found by Bertin et al. (17). Since the first report of *CARD9* mutation in patients with chronic cutaneous and mucosal candidiasis in 2009 (18), there have been successive reports indicating that *CARD9* mutations can significantly increase the susceptibility to a variety of fungi. *CARD9* can effectively integrate the recognition signals of various natural immune receptors and plays an important role in antifungal immunity. As an important mediator of C-type lectin receptor and other receptor pathways, it activates inflammatory signalling pathways such as nuclear factor κB, and then activates downstream signalling molecules to promote the production of inflammatory cytokines (19). *CARD9* mutations are autosomal recessive, and homozygous mutations are the most pathogenic; complex heterozygous mutations have also been reported. At present, it is known that patients with *CARD9* gene mutation are prone to fungal infection, and the common pathogens are *Candida*, *Aspergillus*, and *dermatophytes* (20–22).There have been no reports of *CARD9* mutation in patients with TM infection.

Medical whole exon sequencing showed a complex heterozygous *CARD9* mutation, one of which was c.1118G>C p.R373P, which has been reported in the literature. The findings demonstrate that through impaired phagocytic killing, human CARD9 deficiency results in a selective defect in the host defence against invasive infection (23). C.610C>T p.R204C was a new and previously unreported mutation site, and its pathogenicity was predicted using software. Further tracing of the family revealed that the mutation c.1118G>C p.R373P was inherited from the patient’s father, who in turn inherited it from the paternal grandmother. C.610C>T p.R204C was inherited from the patient’s mother. From a genetic perspective, we speculate that the patient’s second sister may have also had a complex heterozygous *CARD9* mutation, dying as a result of TM infection. The cytokine analysis of the child showed no significant increase in IL-6 and TNF, suggesting that the presence of a complex heterozygous *CARD9* mutation may lead to decreased antifungal immunity and a susceptibility to TM infection.

In conclusion, clinicians should be alert to the possibility of primary immunodeficiency when TM infection occurs in HIV-negative individuals from endemic areas. mNGS and immunodeficiency related gene test should be completed as soon as possible for suspected patients.

## Ethics Statement

Wri**t**ten informed consent was obtained from patients’ parents for publication of this case report and any potentially identifying information. The work was exempt from the ethics committee review/approval.

## Author Contributions

All authors listed have made a substantial, direct, and intellectual contribution to the work, and approved it for publication.

## Funding

The study was supported by Guangdong Basic and Applied Basic Research Foundation (2020A1515010184).

## Conflict of Interest

The authors declare that the research was conducted in the absence of any commercial or financial relationships that could be construed as a potential conflict of interest.
